# Mitochondrial Dysfunction Is Involved in the Toxic Activity of Boric Acid against *Saprolegnia*

**DOI:** 10.1371/journal.pone.0110343

**Published:** 2014-10-29

**Authors:** Shimaa E. Ali, Even Thoen, Øystein Evensen, Jannicke Wiik-Nielsen, Amr A. A. Gamil, Ida Skaar

**Affiliations:** 1 Norwegian Veterinary Institute, Oslo, Norway; 2 Norwegian University of Life Sciences, Oslo, Norway; Universidad Pablo de Olavide, Centro Andaluz de Biología del Desarrollo-CSIC, Spain

## Abstract

There has been a significant increase in the incidence of *Saprolegnia* infections over the past decades, especially after the banning of malachite green. Very often these infections are associated with high economic losses in salmonid farms and hatcheries. The use of boric acid to control the disease has been investigated recently both under *in vitro* and *in vivo* conditions, however its possible mode of action against fish pathogenic *Saprolegnia* is not known. In this study, we have explored the transformation in *Saprolegnia* spores/hyphae after exposure to boric acid (1 g/L) over a period 4–24 h post treatment. Using transmission electron microscopy (TEM), early changes in *Saprolegnia* spores were detected. Mitochondrial degeneration was the most obvious sign observed following 4 h treatment in about 20% of randomly selected spores. We also investigated the effect of the treatment on nuclear division, mitochondrial activity and function using confocal laser scanning microscopy (CLSM). Fluorescence microscopy was also used to test the effect of treatment on mitochondrial membrane potential and formation of reactive oxygen species. Additionally, the viability and proliferation of treated spores that correlated to mitochondrial enzymatic activity were tested using an MTS assay. All obtained data pointed towards changes in the mitochondrial structure, membrane potential and enzymatic activity following treatment. We have found that boric acid has no effect on the integrity of membranes of *Saprolegnia* spores at concentrations tested. It is therefore likely that mitochondrial dysfunction is involved in the toxic activity of boric acid against *Saprolegnia* spp.

## Introduction

*Saprolegniosis* is a common problem in cultured freshwater fish. The disease is caused by species in the genus *Saprolegnia,* which belongs to the class Oomycetes. Infection can cause significant mortality among developing salmonid eggs and fry and contributes to severe economic losses [1]. Moreover, this problem can persist in the presence of treatment due to biofilms as described recently [2]. Outbreaks of saprolegniosis in aquaculture have increased after the banning of malachite green [3]. Thus, there is an urgent need to find efficacious alternatives for prophylaxis and treatment against this pathogen. Boron is omnipresent in nature and forms inorganic borate compounds when it binds to oxygen [4]. Although boron and its compounds have been identified as growth stimulators for fish [5], [6] in a relatively high concentration, they can also be used to control bacterial and fungal infection. Boric acid for example has been used as an effective and safe candidate for controlling yeast and fungal infections in humans and plants [7]–[9]. Generally, the exact mode of action of BA is still not fully known. However, some studies indicated that mitochondrial degeneration and consequent inhibition of oxidative metabolism were the most prominent features observed following boric acid treatment [7], [10]. A recent study suggested that boric acid could be used effectively to limit saprolegniosis in Atlantic salmon eyed eggs and yolk sac fry [11]. Therefore, we aimed to reveal the mechanisms underlying the activity of the boric acid in the control of saprolegniosis. The effect of boric acid on nuclear division in *Saprolegnia* spores/hypha, mitochondrial activity, distribution and cell membrane integrity was studied by means of transmission electron microscopy (TEM), confocal laser scanning microscopy (CLSM) and fluorescence microscopy. The general metabolism, viability and proliferation of treated spores were also tested using a MTS tetrazolium compound (MTS assay).

## Materials and Methods

### Chemical treatments

Boric acid (BA), H_3_BO_3_, M 61.83 g/mol (Merck) was used as a source of borate for the *in vitro* treatment of *Saprolegnia* spores/hyphae. Boric acid was dissolved in sterilized aquarium water (SAW) to give a concentration of 1 g/L. Bronopol (Pyceze, Novartis) was included as a positive treatment control for all tested candidates (100 mg/L). Samples with no treatment diluted in SAW were included in all analysis (non-treated water control).

### *Saprolegnia* isolates and zoospore production

To investigate the internal changes in *Saprolegnia* spores/hyphae elicited by boric acid treatment, three strains of *Saprolegnia* spp. were used, *S. parasitica* (VIO 2736 and VIO 5730) and *S. diclina* (VIO 2739). To produce zoospores, the method described by Stueland et al. [12] was followed. Briefly, *Saprolegnia* hyphae were excised from colonized GY agar plates and incubated in GY broth at 15°C for 2 days to obtain further hyphal growth. Subsequently, bundles of these young hyphae were washed twice in SAW, transferred to two glass bottles containing one liter of SAW and incubated at 21°C for 24 h to allow extensive zoospore production.

### Alterations in *Saprolegnia* spores following boric acid treatment using transmission electron microscopy (TEM)

The early morphological changes in BA treated *Saprolegnia* spores and non-treated controls were investigated by TEM. A similar method to that described by Shi et al (2011) was used. Briefly, spores were concentrated by centrifugation at 16,000 × g for 15min. Boric acid treatment was applied at 1 g/L for 4 h. Treated spores and non-treated controls were fixed in a solution of 1.25% glutaraldehyde and 2% paraformaldehyde in SAW. The pellets were rinsed thoroughly in 0.1M sodium cacodylate buffer (SCB). Gels of 1–2 mm^3^ were prepared by adding 3% low gelling temperature agarose in distilled water to the pellet. The gels were post-fixed with 1% osmium tetroxide in SCB for 2 h at room temperature. After thorough rinsing with SCB, the gels were dehydrated with 10 min stages in an ascending ethanol series (50–100%). The samples were embedded in LR White resin. Ultrathin sections were obtained with a Leica EM UC6 Ultramicrotome and stained by 4% uranyl acetate and 1% potassium permanganate for 10 min. Mitochondrial changes in the spores were detected using a FEI Morgagni 268 transmission electron microscope at 80 kV.

### Effect of boric acid on the nuclear division and germination of *Saprolegnia* spores

The fluorescent dye 4′,6-diamidino-2-phenylindole (DAPI) dilactate (Invitrogen) was used to observe nuclear changes and division in BA treated *Saprolegnia* spores and controls. DAPI is a nucleic acid specific dye [13]. It has been used to visualize nuclear changes in treated yeast [14] and fungi [8]. A stock solution was prepared by dissolving 10 mg of the probe in 2 mL of deionized water (dH_2_O) to have final concentration of 5 mg/ml. Freshly harvested *Saprolegnia* spores were exposed to 1 g/L BA in the presence of 0.5% Glucose-yeast broth (GY broth) [15]. Treated spores and non-treated controls (spores in SAW) were incubated for 2, 4, 6 and 8 h at 15°C. Later on, spores were concentrated by centrifugation, 16,000 × g for 10 min, fixed in 70% ethanol and incubated overnight at 4°C. Spores were centrifuged again and washed with SAW to remove ethanol. Spores were then seeded into 4-well chambered slides, and incubated in a staining solution of 25 nM 4′,6-diamidino-2-phenylindole (DAPI) [16]. Samples were examined by confocal laser scanning microscopy (CLSM) (Zeiss LSM710) with a 405 nm laser.

### Effect of boric acid on mitochondrial activity, distribution and membrane potential using confocal laser scanning microscopy (CLSM)

MitoTracker Red CMTMRos probes (50 µg; Invitrogen) were used to assess the mitochondrial changes in spores and hyphae after BA treatment. MitoTracker is a mitochondrion-specific stain and its accumulation depends on the membrane potential.

Stock solution was prepared by dissolving 50 µg of the stain in anhydrous dimethylsulfoxide (DMSO) (Invitrogen) to obtain 1 mM concentration. Stock solution was diluted in GY broth to give a 50 nM MitoTracker working solution. *Saprolegnia* spores were treated with BA 1 g/L for 4, 12 and 24 h and suspended in 50 nM MitoTracker solution. After 10–15 min incubation in the dark, excess stain was washed out and the spores were seeded in chambered slides and examined directly with CLSM, generating single plane images using a 561 nm laser line to excite the MitoTracker Red. To follow the changes within treated *Saprolegnia* hyphae, mycelia were prepared as follows: *Saprolegnia* cultures grown on agar plugs were incubated in GY broth for more mycelial growth. The agar plug was removed from the broth and re-suspended in BA (1 g/L). The same protocol as described for spores was applied for mycelia. Stained, treated mycelia were examined directly using ordinary microscopic slides. Spores/mycelia in water served as non-treated control. The images were generated on a Zeiss LSM 710 confocal microscope using a 561 nm laser to excite the Mitotracker red, through a 63x plan apochromat oil immersion objective. The fluorescence intensity was measured in spores/hyphae, following 24 h treatment, as spot measurements (25/hyphal apex, spot size: approx. 850 pixels) within the hyphal apex or spore. Measurements were performed using ZEN software (Zeiss). Mitotracker red is a live-cell dye, and uptake of the dye is expected to be higher in intact mitochondria than in compromised ones. Fluorescence intensity was thus used as a quantitative indicator of damage, alongside the visual difference in distribution.

### Mitochondrial membrane potential and reactive oxygen species (ROS) using fluorescence microscopy

The cell permeating dye tetramethyl rhodamine, ethyl ester (TMRE) 1 mM in DMSO (abcam) was used together with dichlorofluorescein diacetate (DCFDA) cellular reactive oxygen species detection assay kit 20 mM (abcam). TMRE dye was used for labeling mitochondrial membrane potential and function [17] and DCFDA as an indicator for the reactive oxygen species (ROS) activity within boric acid treated hyphae and the non-treated control. DCFDA is able to diffuse into the cell and is de-acetylated by cellular esterases to a non-fluorescent compound, which is later oxidized by ROS into 2′,7′–dichlorofluorescin (DCF). DCF is a highly fluorescent compound which can be detected by fluorescence microscopy. Briefly, hyphae on agar plugs grown in GY broth were exposed to BA treatment for 24 h and those without treatment were kept as a control. Preparations were suspended in 300 µl of dye (50 µl of TMRE working solution (100 µm) and 250 µl of DCFDA diluted in SAW (10 µm)). They were then incubated in the dark for 45–60 min before the excess unbound dyes were washed out and examined directly with fluorescence microscopy (Olympus IX81 motorized inverted microscope).

### Assessing mitochondrial activity and viability of boric acid treated *Saprolegnia* spores using MTS assay

CellTiter 96 aqueous one solution cell proliferation assay (Promega) was used. This assay involves the reduction of tetrazolium compound [3-(4,5-dimethylthiazol-2-yl)-5-(3-carboxymethoxyphenyl)-2-(4-sulfophenyl)-2H-tetrazolium, inner salt; MTS] by viable cells to form formazan products from MTS reduction. The reaction occurs when mitochondrial enzymes are active (Mitochondrial dehydrogenase) [18], and could be used as an indicator for the metabolic rate of the mitochondria [19]. Briefly, 96-well plates were seeded with 100 µl of *Saprolegnia* spores in 10% GY broth. 50 µl of boric acid was added to each tested well (except controls) to have a final concentration of 1 g/L. Bronopol was used as a positive treatment control and SAW as a negative control. MTS reagent was added to all tested wells and controls (50 µl/well). Wells with the MTS reagent without spores were used as a background control. The plates were incubated at 15–20°C. Reading was first recorded on 96-well plate reader following 4 h incubation, then after 8 and 24 h of treatment application. The amount of the formazan product was measured by the absorbance at 490 nm. The MTS reduction is directly proportional to the number and activity of the living spores. Background absorbance was also measured. Spore viability was calculated as follows: % viability = 100 × (O.D_490_ value for the sample – mean background O.D_490_)/(mean O.D_490_ for non-treated water control – mean background O.D_490_).

### Effect of boric acid on the integrity of *Saprolegnia* spore membranes

The direct effect of boric acid on *Saprolegnia* spore membranes was assessed by viability staining. Propidium iodide (PI) 1.0 mg / mL solution in water (Invitrogen) was used to test the membrane integrity and viability of treated spores. It is based on the principle that live cells with intact membranes are normally able to exclude dyes that easily penetrate damaged or necrotic cells [20]. PI has been widely used in different experimental models to determine the cell viability [21] and for staining the cell walls of fixed plant material [22]. It was also used to distinguish live *Saprolegnia* hyphae from dead ones in biofilm [2]. *Saprolegnia* spores with 1% GY broth were distributed in chambered slides and BA was added to have a concentration of 1 g/L. Spores treated with bronopol were used as positive control and those in water were used as non-treated control. Following 24 h incubation, 2–4 drops of PI (2 µg/ml) were added. The fluorescent nucleic acid dye SYTO 9 (Invitrogen) was used as a counter stain to visualize live spores/hyphae. Slides were kept in the dark for 5 min prior to examination with fluorescence microscopy.

### Statistics

The differences in viability between boric acid, bronopol and non-treated control were assessed by multiple t-test using GraphPad Prism version 6.00 for Windows, GraphPad Software, La Jolla California USA, www.graphpad.com, and considered significant at p<0.05.

## Results

### Alterations in *Saprolegnia* spores following boric acid treatment using transmission electron microscopy (TEM)

**Figure 1:**
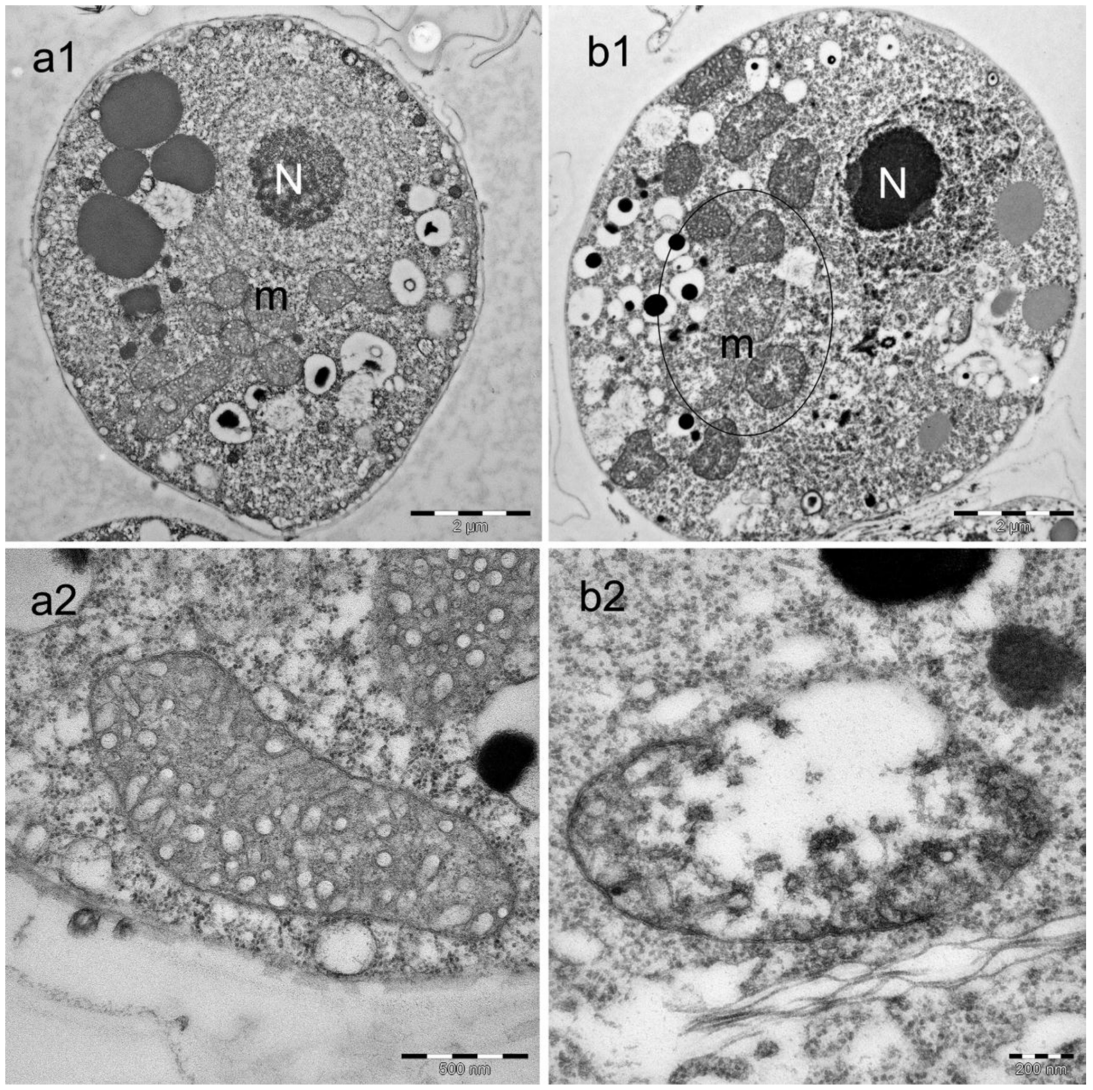
Alterations in *Saprolegnia* spores following boric acid treatment. Transmission electron microscopy image of an untreated *Saprolegnia* spore (a) and a *Saprolegnia* spore treated with boric acid (1 g/L) for 4 h (b). Normal, well defined mitochondrial structure is seen in the non-treated spores (a1 and a2) compared to the spore that has been exposed to boric acid (b1 and b2). Different degrees of degenerative changes were observed in the mitochondria of the treated spore (circle). The condensed nucleus (N) with disintegrated nuclear membrane is seen in the treated spore (b1), but this was not a consistent finding and seen only in a few spores.

**Figure 2:**
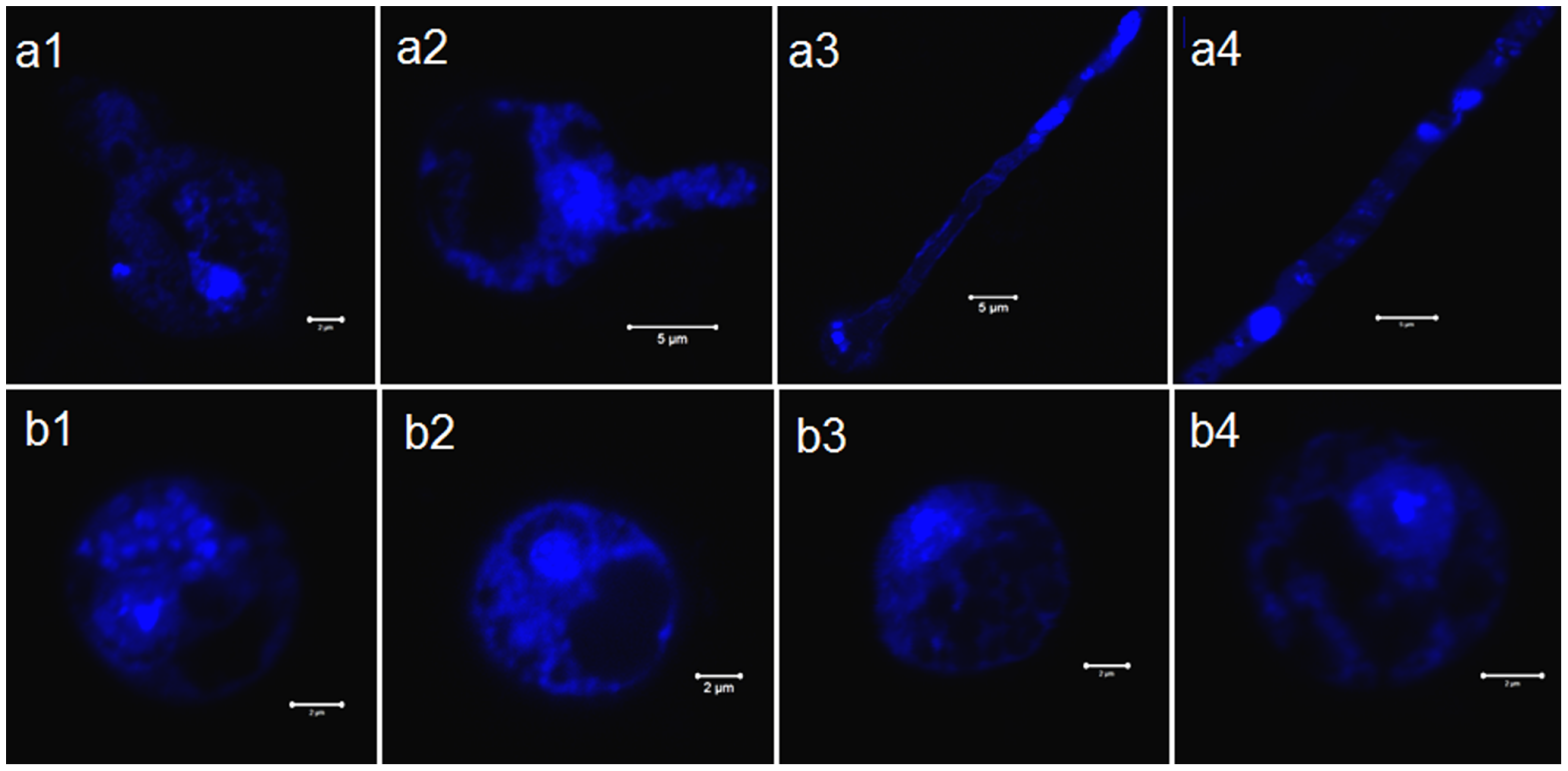
Effect of boric acid on the nuclear division and germination of *Saprolegnia* spores. Confocal laser scanning microscopy images of *Saprolegnia* spores stained with the nucleic acid dye DAPI. a1–a4) Spore germination in non-treated water control group. Note the movement of the nucleus towards the newly developing germ tube following 2 and 4 h incubation (a1 and a2). Development of multinuclear hyphae indicating growth and viability is shown in image a3 and a4. b1–b4) Gradual reduction of fluorescence intensity of *Saprolegnia* spores treated with boric acid following 2, 4, 6 and 8 h of incubation, b1, b2, b3 and b4 respectively. No nuclear division was observed in the treated group.

The majority of treated spores showed mild alterations in mitochondrial morphology and structure at 4 h after BA treatment. Mitochondrial damage was observed in 20% of randomly selected BA treated spores compared to the non-treated water control spores (Fig. 1). The changes included swelling of the mitochondria with loss of cristae and tubules (Fig. 1b-2). There was a trend towards spores appearing with condensation of the nucleus with disintegration of the nuclear membrane, however, this was not a consistent finding (Fig. 1).

### Effect of boric acid on the nuclear division and germination of *Saprolegnia* spores

**Figure 3:**
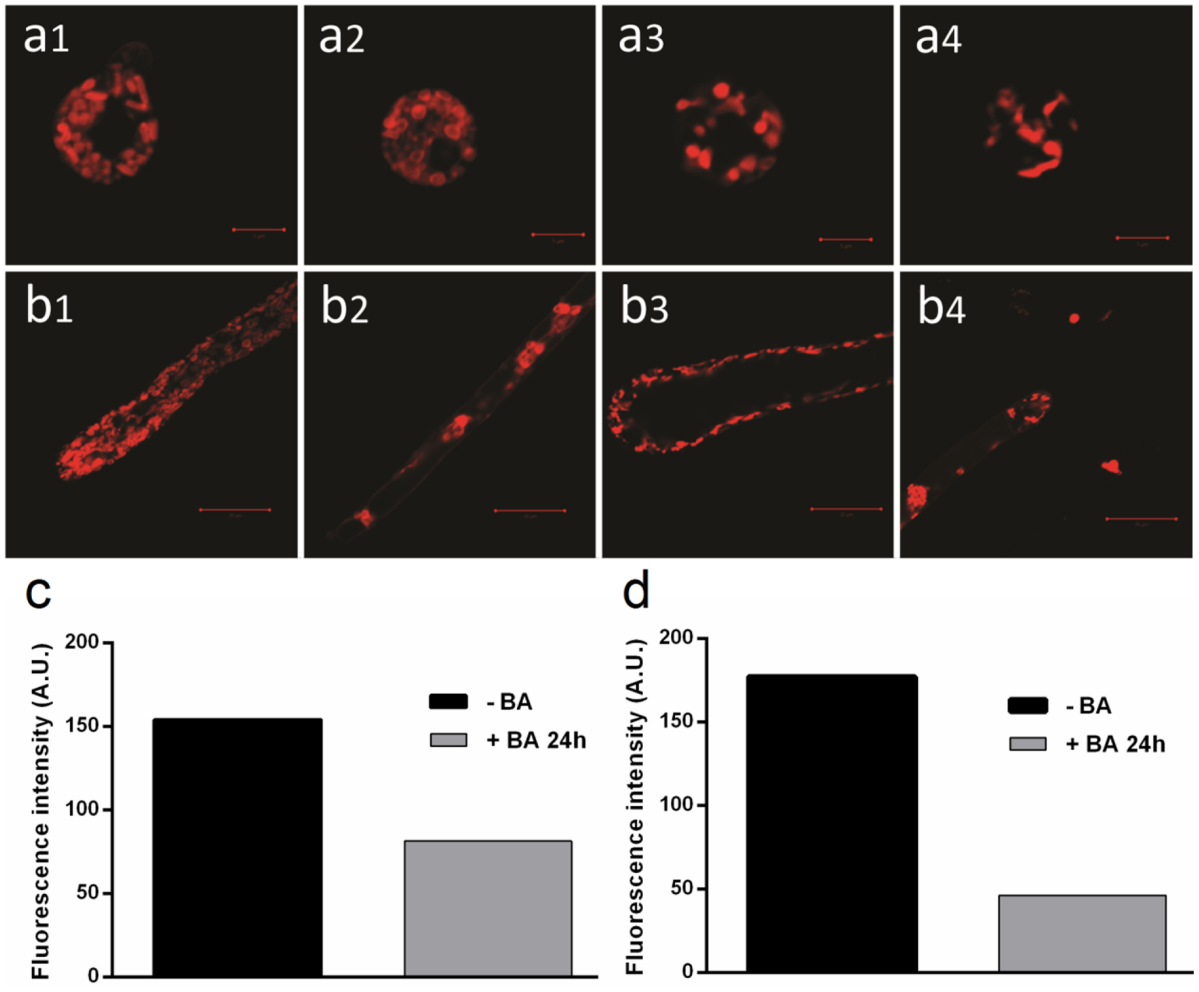
Effect of boric acid on *Saprolegnia* mitochondria using confocal laser scanning microscopy. Confocal laser scanning microscopy showing the effect of the boric acid on *Saprolegnia* spore (a) and hyphal (b) mitochondrial activity using MitoTracker Red. a1) Accumulation of the stain in the non-treated control. Gradual reduction in the number of mitochondria in treated spores 4 (a2), 12 (a3), and 24 (a4) hours after boric acid treatment. b1) *Saprolegnia* hyphae with densely distributed mitochondria indicating high activity in the non-treated control. Pronounced degradation of hyphal mitochondria 4 (b2), 12 (b3), and 24 (b4) hours post boric acid treatment. Figure 3 c and d show the average fluorescence intensity of BA treated *Saprolegnia* spores (c) and hyphae (d) compared to the non-treated control following 24 h exposure.

**Figure 4:**
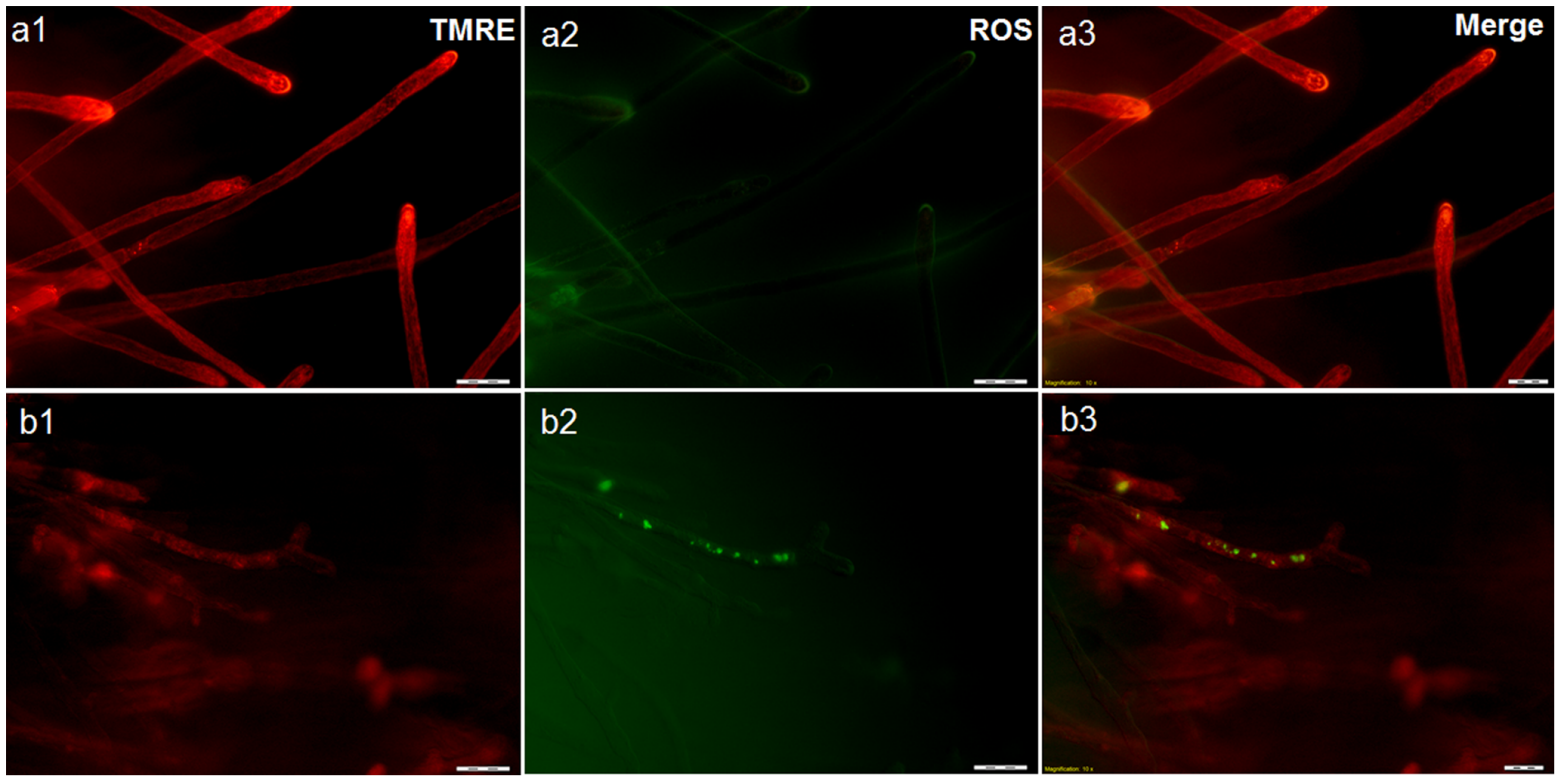
Effect of boric acid on *Saprolegnia* mitochondria using fluorescence microscopy. Fluorescence microscope image showing the concentration of tetramethyl rhodamine (TMRE) staining in healthy non-treated *Saprolegnia* hyphae (a1) compared to boric acid treated hyphae (b1) where the depolarized mitochondria exhibit reduced red fluorescence. Figure (a2) is showing ROS level in the non-treated control compared to treated *Saprolegnia* hyphae (b2). TMRE and ROS staining are merged in a3 and b3.

The absence of nuclear division and germination was the most prominent features observed in BA treated spores (Fig. 2b) compared to non-treated water control (Fig. 2a). However, no fixed morphological pattern for the nuclear division was exhibited by those in the non-treated water control group. Some nuclei were able to divide inside the cyst body, while the majority migrated with the whole nucleic acid contents toward the germ tube leaving an almost empty cyst body structure. Other observations in the treated spores were an increase in the vacuolization and decrease in the fluorescence intensity of the extra-nuclear DNA (Fig. 2 b-3 and b-4), apparently due to the reduction in mitochondrial activity as the active mitochondria have a small amount of their own DNA.

### Effect of boric acid on mitochondrial activity, distribution and membrane potential using confocal laser scanning microscopy (CLSM)

*Saprolegnia* spores treated with BA for 4 h (Fig. 3a-2) showed minor mitochondrial changes compared to the non-treated water control (Fig. 3a-1). There appeared a significant reduction in the spore mitochondrial numbers observed at 12 and 24 h following BA treatment (Fig. 3a-3 and a-4). Treated hyphae showed reduction in the number of hyphal apex mitochondria following 4 h exposure (Fig. 3b-2). After 12 h treatment, swollen hyphal apex and changes in the pattern of mitochondrial distribution were demonstrated (Fig. 3b-3). Different vacuolar forms without mitochondrial activity were formed throughout the apical part of the hyphae 24h post initiation of treatment (Fig. 3b-4). In contrast, the mitochondria in the non-treated control showed dense red-colored cylindrical and round structures concentrated in the hyphal apex and distributed all over the hyphae (Fig. 3b-1). Figure 3 also shows the difference in fluorescence intensity, in BA treated *Saprolegnia* spores (c) and hyphae (d) compared to the non-treated control following 24 h exposure.

### Effect of boric acid on mitochondrial membrane potential and reactive oxygen species (ROS) using fluorescence microscopy

The drop in the fluorescence intensity and distribution of TMRE dye were the main differences observed in BA treated *Saprolegnia* hyphae (Fig. 4b-1) compared to the non-treated water control (Fig. 4 a-1). The dye accumulation in the hyphal apex of the non-treated control is an indication for their mitochondrial activity. ROS accumulation was detected in some of the BA treated hyphae (Fig. 4b-2). However, it was also observed in hyphae in the non-treated control with weak mitochondrial activity.

### Assessing the metabolic activity and viability of treated spores using the MTS assay

**Figure 5:**
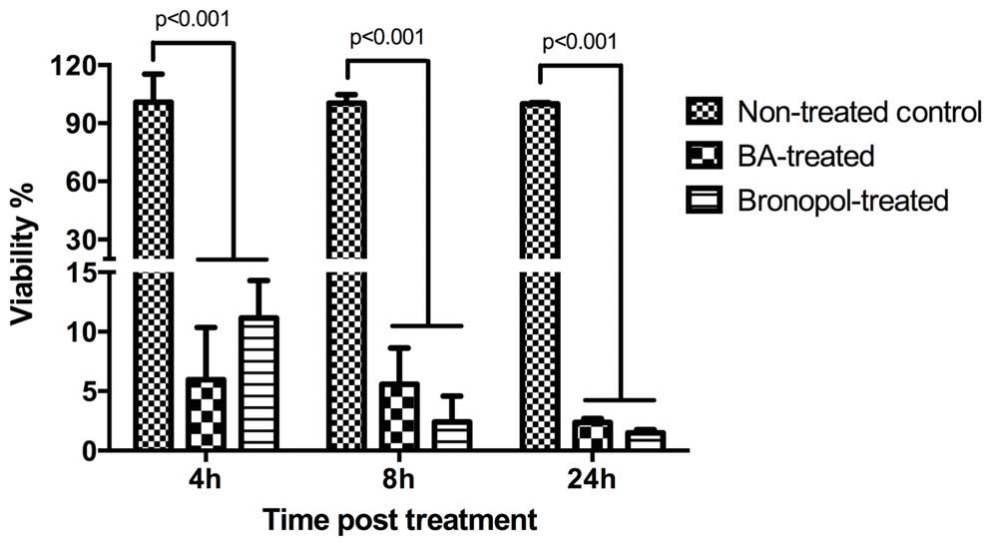
Viability of treated *Saprolegnia* spores at different time post treatment. Mitochondrial activity and viability in *Saprolegnia* spores following boric acid (1 g/L) and bronopol (100 mg/L) treatment was compared to non-treated control using the MTS assay. The diagram shows the percent of viable spores relative to the non-treated ones calculated as described in the methodology section. Spores viability was significant reduced (p<0.001) in BA and bronopol treated samples at all time points (4–24 h) relative to non-treated control.

The spores’ viability was significantly reduced after BA and bronopol treatment (Figure 5). At 4 h post treatment, 6% of the spores were found viable after BA treatment compared to 11% in bronopol-treated ones. The percent of viable spores remained unchanged at 8 h post treatment in BA treated groups (6%) while dropped to less than 5% in the bronopol treated samples. By 24 h post treatment, 2.4 and 1.5% of the spores were viable in BA and bronopol treated groups, respectively. BA and bronopol groups were significantly lower at all-time points while there was no difference between BA and bronopol (Figure 5).

### Effect of boric acid on the integrity of *Saprolegnia* spore membranes

**Figure 6:**
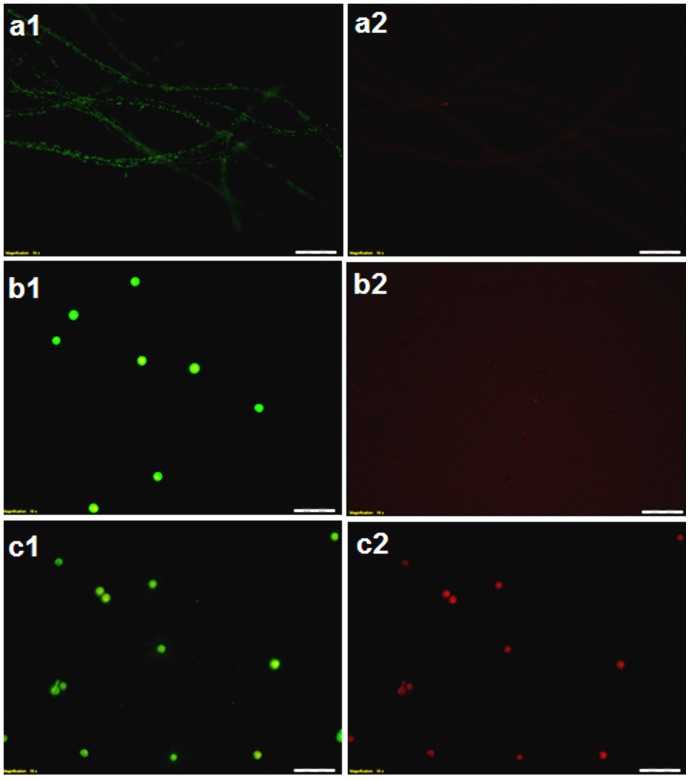
Effect of boric acid on the integrity of *Saprolegnia* spore membranes. Fluorescence microscopy analysis of Propidium Iodide (PI) uptake by *Saprolegnia* spores. a) non-treated spores kept in water, were able to germinate and to form hyphae that only flourecent green with SYTO 9 (a1) without PI uptake (a2). b) Boric acid treated spores, neither germinate (b1) nor absorb the PI dye (b2). c) Non-viable, bronopol treated *Saprolegnia* spores showing uptake of SYTO 9 (c1) and PI dye (c2).

Fluorescence microscopy revealed that *Saprolegnia* spores treated with BA neither absorbed the propidium iodide dye nor germinated, which indicate the integrity of plasma membrane was maintained as shown by staining with SYTO 9 dye (Fig. 6b). In contrast, bronopol treated spores fluoresced red and green as the propidium iodide dye was able to diffuse into their necrotic walls (Fig. 6c). Spores kept in water were able to germinate and to form hyphae that only fluoresce green (Fig. 6a–1).

## Discussion

This study indicates that boric acid has a toxic effect on *Saprolegnia* spore germination and hyphal growth under *in vitro* condition. The degeneration of mitochondria or the inhibition of its enzymes and metabolism is probably the key for its mode of action.

The results obtained by the transmission electron microscopy following 4 h of exposure, indicated mild alterations in nuclear morphology in some of the BA treated spores (condensed nuclei with fragmentation of their membranes). Additionally, a considerable variation in the mitochondrial structure of BA treated spores compared to the non-treated water control was observed. Mitochondria are essential cellular organelles that play central roles in energy production, metabolism generation, regulation of reactive oxygen species (ROS) and apoptosis [23], [24]. They are also responsible for more than 90% of cellular adenosine triphosphate (ATP) production which is essential for the cell to perform all essential activities for its survival and function [10], [25], [26]. Therefore, compounds able to affect the mitochondrial respiratory chain could be used as growth inhibitors [18], [24], [27]. Hyphal-growing organisms, including *Saprolegnia* usually have a high density of apex-localized mitochondria [29]. They play a role in the high Ca^2+^ gradient in the apex, which is important for the apical growth [28], [29]. The mitochondria localized behind the tip are the ones responsible for the respiratory function and ATP production [29]. Using MitoTracker Red probes and CLSM, boric acid treated hyphae showed considerable variations in the number and distribution of mitochondria localized both in the hyphal apex and through the hyphae. The same result was recorded in the treated *Saprolegnia* spores. The decrease in the mitochondrial density was directly proportional with the exposure time, when different time points were used. The reduction and disorganization in spore/hyphal mitochondria following boric acid treatment is supporting the hypothesis that mitochondrial toxicity might be one of its primary modes of action. Mitochondrial membrane potential (ΔΨm) is critical for maintaining many mitochondrial processes including ATP synthesis and the control of ROS generation; its disturbance could diminish energy production [30]. Thus, changes in ΔΨm were also followed using the fluorescent dye (TMRE). Hyphae from non-treated controls showed intense fluorescence as TMRE dye accumulates in active mitochondria due to their relative negative charge. On the other side, boric acid treated hyphae showed a drop in the fluorescence intensity as the inactive mitochondria with decreased membrane potential fail to sequester TMRE. Reactive oxygen species (ROS) are important signaling molecules normally present in cells, however, their accumulation under pathological conditions leads to oxidative stress. Therefore, ROS with ΔΨm can be used as an indicator of the cell physiological status and the function of the mitochondria [30]. This might explain the high accumulation of ROS in some of the treated hyphae with a lower mitochondrial activity than the non-treated ones.

Regarding the nucleus, no obvious changes were observed on the nuclear morphology of the treated spores examined by TEM. The nuclear mitosis has been described before in *Saprolegnia* spp. [31], thus, the effect of the boric acid on the nuclear division was followed using the nucleic acid specific dye DAPI. Spores kept in water were able to develop germlings that elongated. Almost all the organelle-containing cytoplasm in the cyst body was able to relocate to the developed germlings as described before [32] and the nucleus was able to divide. In contrast, the nuclei of the treated spores failed to divide when they were examined with the CLSM. This effect of the boric acid on the nuclear division might be correlated to a reduction in DNA synthesis as suggested by Ku et al. [33], apparently due to the absence of sufficient energy related to the impairment of normal mitochondrial function.

The metabolic activity and proliferation of treated spores were also investigated using an MTS assay. This assay relies on the metabolism of the MTS reagent into formazan by dehydrogenase enzymes [34]–[36]. It thereby gives an indication on the functional state and integrity of the mitochondria. The significant reduction of the MTS reagent in the non-treated water control group following 24 h incubation indicates their high mitochondrial metabolic and proliferation activity. In contrast, the boric acid treated group has almost similar results as the positive control group treated with bronopol.

The integrity of the membrane of treated *Saprolegnia* spores was interpreted by using a propidium iodide stain. The fact that the treated spores did not take up the dye nor germinate is suggesting that boric acid does not have a direct effect on treated spore membranes at the tested concentration which also suggests that BA could cause slow loss of viability.

All in all our study is explaining the toxic activity of boric acid on *Saprolegnia* by its effect on mitochondrial function and the inhibition of the general metabolism. Future investigations for the possibility of combining boric acid with potential anti-*Saprolegnia* candidates targeting cellular components other than mitochondria should be considered.

## Conclusion

We can conclude that boric acid possess a toxic activity on *Saprolegnia* spores germination and hyphal growth. The disruption of metabolism and impairment of the normal mitochondrial function or some of its enzymes are probably included in its primary mode of action.
